# Diagnostic Challenge of Xanthogranulomatous Salpingo‐Oophoritis in an 8‐Year‐Old Girl in a Low Resource‐Setting: A Rare Case Report

**DOI:** 10.1002/ccr3.71124

**Published:** 2025-10-16

**Authors:** John Lugata, Kristen Riley, Laetitia Makower, Baraka Shao, Tecla Lyamuya, Alex Mremi

**Affiliations:** ^1^ Department of Obstetrics and Gynecology Kilimanjaro Christian Medical Centre Moshi Tanzania; ^2^ School of Medicine KCMC University Moshi Tanzania; ^3^ Weill Cornell Medical College, Weill Cornell Medicine New York New York USA; ^4^ School of Clinical Medicine University of Cambridge Cambridge UK; ^5^ Department of Pathology Kilimanjaro Christian Medical Centre Moshi Tanzania; ^6^ Kilimanjaro Clinical Research Institute Moshi Tanzania

**Keywords:** diagnostic challenge, neuroblastoma, resource‐constraint settings, school‐aged girl, xanthogranulomatous salpingo‐oophoritis

## Abstract

Xanthogranulomatous oophoritis is an extremely rare, chronic inflammatory condition of the ovary characterized by the replacement of normal ovarian tissue with lipid‐laden macrophages, plasma cells, and lymphocytes. When the genital tract is involved, this most commonly affects the endometrium, whereas ovarian and fallopian tube involvement is rare. It is often misdiagnosed due to its nonspecific clinical and radiological features, which can mimic neoplastic or infectious pathologies. This case report highlights a rare presentation in a pediatric patient and emphasizes the diagnostic challenges encountered, especially in low‐resource settings. To the best of our knowledge, this is the first reported case of its kind in Tanzania. We report a rare case of an 8‐year‐old girl who presented to our specialty hospital in Northern Tanzania with a history of progressive abdominal distension for 8 months. An abdominal CT scan with contrast revealed a large, heterogeneous, enhancing retroperitoneal mass with central necrosis, measuring 13 × 13 × 10 cm. The mass displaced the intestines superolaterally, compressed the urinary bladder inferiorly, and displaced the ureters laterally, resulting in bilateral hydroureteronephrosis. It abutted the bilateral iliac arteries and compressed the sigmoid colon. Multiple enlarged para‐aortic, periportal, and mesorectal lymph nodes were visualized, the largest measuring 2 × 2 cm. There was minimal free intraperitoneal fluid, and a CT scan of the chest with contrast revealed two micronodules in the anterior segment of the right upper lobe of the lung. Radiological findings were suggestive of a retroperitoneal tumor with pulmonary metastases. Based on clinical and radiological findings, a working diagnosis of Neuroblastoma was made. The patient was initiated on a standard Neuroblastoma chemotherapy protocol and received three cycles, which resulted in a significant reduction in tumor size. Subsequently, she underwent an exploratory laparotomy, during which a left salpingo‐oophorectomy was performed. Postoperative recovery was uneventful and the patient was discharged in stable condition. The final histopathological report showed xanthogranulomatous salpingo‐oophoritis, thus excluding the initial diagnosis of malignancy.

**Summary**

- Xanthogranulomatous salpingo‐oophoritis is an exceedingly rare and often misdiagnosed chronic inflammatory condition, especially in pediatric patients.
- Its clinical, radiological, and biochemical presentation can closely mimic malignant retroperitoneal or ovarian tumors, leading to potentially unnecessary administration of chemotherapy or extensive surgical procedures.
- Awareness of this entity among clinicians can prevent mismanagement and significantly improve patient outcomes.

## 1 Introduction

Xanthogranulomatous inflammation is a rare benign inflammatory condition in which normal tissue is replaced by lipid‐laden foamy macrophages and other inflammatory cells including lymphocytes, plasma cells, neutrophils, and giant cells [1]. Xanthogranulomatous inflammation is most commonly seen in pyelonephritis; however, it has more recently been described in an array of other locations including the bladder, bone, lung, intestines, adrenal gland, endometrium, vagina, fallopian tubes, and testis [2]. Given the rarity of the condition, the precise pathophysiology of xanthogranulomatous inflammation is not well understood. It is thought to begin with an inciting event, such as infection, obstruction, or trauma, which is then followed by an inflammatory response in which neutrophils, lymphocytes, plasma cells, and macrophages are recruited to the site [3]. This then leads to granulomatous tissue formation and ongoing inflammation, eventually resulting in a mass‐like presentation. Xanthogranulomatous inflammation is most commonly identified in the kidney and gallbladder. However, rare cases of the condition have been reported in the gastrointestinal tract, the skin, and the female genital tract [4].

Historically, cases of xanthogranulomatous inflammation involving the female genital tract most commonly affected the endometrium, with adnexal involvement being exceedingly rare. However, in recent years, more cases of xanthogranulomatous inflammation involving the ovaries and fallopian tubes have been reported [5]. A 2023 single‐institution review of 40 cases of xanthogranulomatous inflammation of the female genital tract identified that almost 90% of cases involved the adnexa [6]. A separate case series published in 2024 identified seven cases involving the adnexa, with two cases of oophoritis and five cases of oophoritis with salpingitis [7].

Xanthogranulomatous oophoritis often presents with abdominal pain and an abdominal mass but can also present with other symptoms, including vaginal bleeding, fever, fatigue, and anemia. Given its overlapping presentation with ovarian neoplasms, xanthogranulomatous oophoritis is often mistaken for malignancy, leading to diagnostic challenges and potential overtreatment [5]. Xanthogranulomatous oophoritis is exceedingly rare in the pediatric population, with most cases presenting in adults ages 21 to 75. There has only been one clearly documented case of xanthogranulomatous oophoritis in a pediatric patient. The 2‐year‐old patient presented with abdominal pain and distention, with a large right‐sided abdominal mass identified on imaging studies. Salpingo‐oophorectomy was performed, with histology revealing infiltration of ovarian tissue by sheets of foamy histiocytes, lymphocytes, and plasma cells, as well as areas of necrosis and fibrosis, confirming the diagnosis of xanthogranulomatous oophoritis [8].

The rarity of xanthogranulomatous salpingo‐oophoritis, along with its presentation mimicking that of ovarian malignancies, can present challenges in both diagnosis and management. Furthermore, given the limited number of reported cases of xanthogranulomatous salpingo‐oophoritis, particularly in the pediatric population, each new case contributes valuable insights to the presentation, diagnosis, and management of this rare condition. This case report describes a rare occurrence of xanthogranulomatous salpingo‐oophoritis in a pediatric patient in Tanzania, highlighting the diagnostic challenges of this condition, particularly in a low‐resource setting.

## 2 Case History

An 8‐year‐old girl presented to our outpatient Gynecology clinic in Northern Tanzania with a history of abdominal distension that started gradually 8 months ago, as per the history narrated by her mother. It was associated with intermittent abdominal pain, episodes of vomiting, nausea, early satiety, changes in bowel habits, increased urinary frequency and urgency, recurrent fevers, night sweats, and significant weight loss. However, she reported no history of diarrhea, headache, coughing, difficulty in breathing, or vaginal bleeding. Her past medical and family history was unremarkable.

On examination, she looked unwell, cachexic, and pale. However, she was conscious, not dehydrated, and afebrile with stable vitals. She weighed 21.8 kg and was 128.8 cm in height, with an abdominal circumference of 58 cm at the level of the umbilicus, with no lower limb pitting edema. The abdomen was distended, with a palpable mass originating from the hypogastric region and extending to the umbilicus, measuring approximately 20 × 18 cm, which was tender on palpation. Additionally, bilateral submandibular and inguinal lymphadenopathy was noted. The examination of other systems was unremarkable. Based on radiological and laboratory findings, and the patient's initial clinical presentation, the leading differential diagnoses included tuberculosis, neuroblastoma, yolk sac tumor, dysgerminoma, and mixed germ cell tumor.

## 3 Methods

**FIGURE 1:**
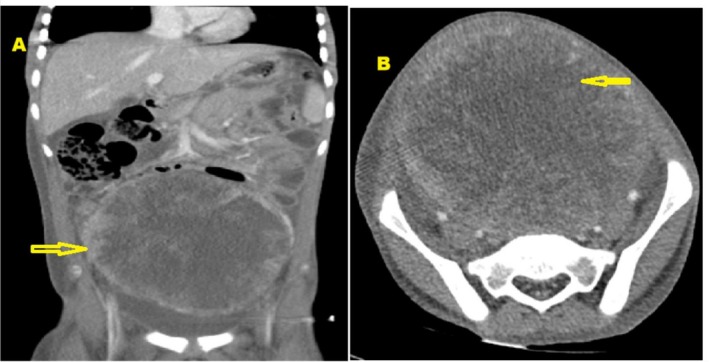
Abdominal CT scan with contrast (A) coronal and (B) axial view revealed a large, heterogeneous, enhancing retroperitoneal mass with central necrosis, measuring 13 × 13 × 10 cm. The mass displaced the intestines superolaterally, compressed the urinary bladder inferiorly, and displaced the ureters laterally, resulting in bilateral hydroureteronephrosis.

An abdominal CT scan with contrast revealed a large, heterogeneous, enhancing retroperitoneal mass with central necrosis, measuring 13 × 13 × 10 cm (Figure 1). The mass displaced the intestines superolaterally, compressed the urinary bladder inferiorly, and displaced the ureters laterally, resulting in bilateral hydroureteronephrosis. It abutted the bilateral iliac arteries and compressed the sigmoid colon. Multiple enlarged para‐aortic, periportal, and mesorectal lymph nodes were visualized, the largest measuring 2 × 2 cm. There was minimal free intraperitoneal fluid, and the CT scan of the chest with contrast revealed two micronodules in the anterior segment of the right upper lobe of the lung. Radiological findings were suggestive of a retroperitoneal tumor with pulmonary metastases. Cancer antigen 125 (CA‐125), βHCG, and carcinoembryonic antigen (CEA) tumor markers were within normal limits. However, alpha fetal protein (AFP) was elevated at 91,973 IU/mL (0.000–6.050). A complete blood count revealed leukocytosis of 14 × 10^9^/L (4.00–11.00), hemoglobin level of 7 g/dL (10–15.5 in ages 6–18), blood group was O positive, albumin was low at 17 L (35–52), Lactate Dehydrogenase (LDH) was elevated at 1139 U/L (240.00–480.00), uric acid was low at 118 μmol/L (142.80–339.20), and serum ferritin was elevated at 866 μg/L; liver and renal function tests were all within normal limits. Blood and urine culture revealed no bacteria growth, the gene expert was negative, and the bone marrow biopsy was normal.

**FIGURE 2:**
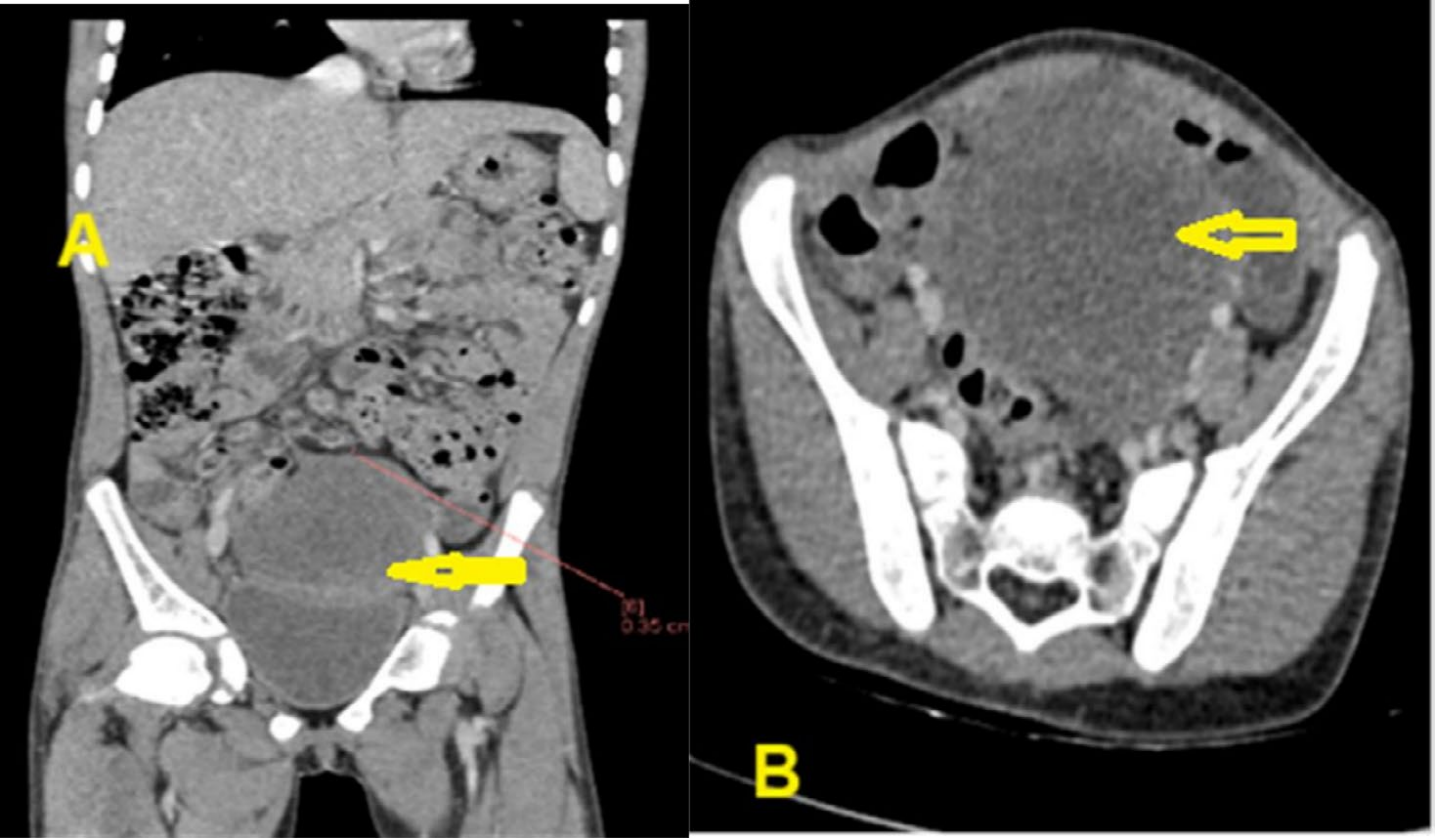
Abdominal CT scan with contrast (A) coronal and (B) axial view revealed significantly reduced in size to 5 × 4 × 5 cm after 5 cycles of chemotherapy, and there was no suspicious nodule for pulmonary metastases.

A multidisciplinary team of gynecologists, oncologists, and pathologists agreed to treat this diagnosis of neuroblastoma with lung lesions. She was started on the neuroblastoma chemotherapy protocol, which included 6 cycles of: Inj. Cyclophosphamide 225 mg, Inj. Carboplatin 492 mg, Inj. Bleomycin 12 mg, Inj. Etoposide 50 mg, Inj. Doxorubicin 26 mg, Inj. Vincristine 2 mg. She received 5 cycles of chemotherapy alongside continued antibiotic therapy, which she tolerated well, and subsequently, the tumor was significantly reduced in size to 5 × 4 × 5 cm (Figure 2). There were no nodules suspicious of pulmonary metastases. The mass demonstrated a marked clinical response compared to its pretreatment status, with significant symptomatic improvement.

## 4 Conclusion and Results

The child was taken for explorative laparotomy, under general anesthesia, with a left lateral tilt to the operating table. The abdomen was opened in layers with an extended umbilical midline incision. Intraoperatively, there was a straw‐colored fluid collection of approximately 10 mL and a firm left tubo‐ovarian mass measuring about 7 × 6 cm. The mass had ruptured on its anterior surface, exposing calcified contents, and was adherent to the right ovary. No bowel involvement was observed. A left salpingo‐ophrectomy was done, and hemostasis was achieved.

**FIGURE 3:**
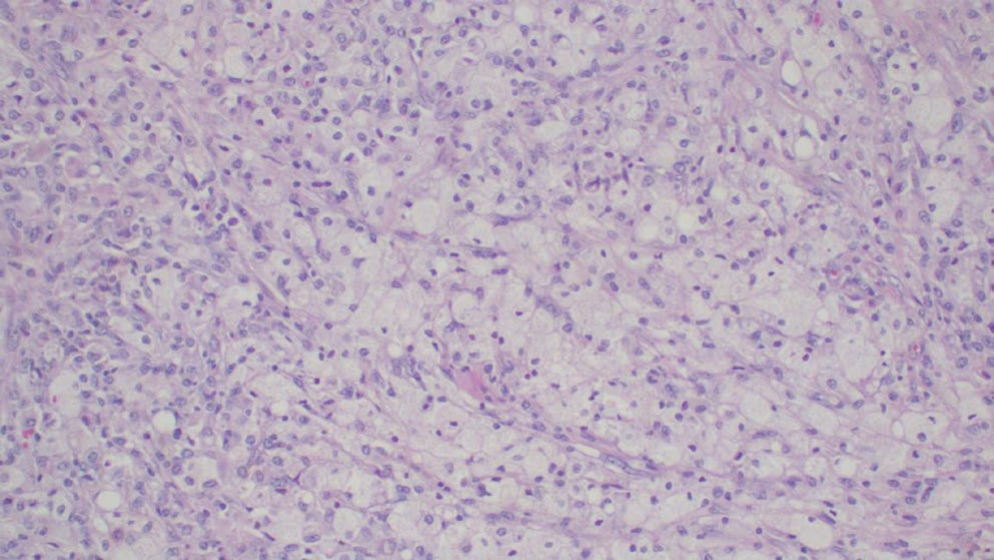
Histopathology of xanthomatous salpingo‐oophoritis highlighting the tissue infiltrated by the dense population of foamy macrophages, histiocytes, and lymphocytes. Hematoxylin and eosin (H&E) staining at 10× original magnification.

**FIGURE 4:**
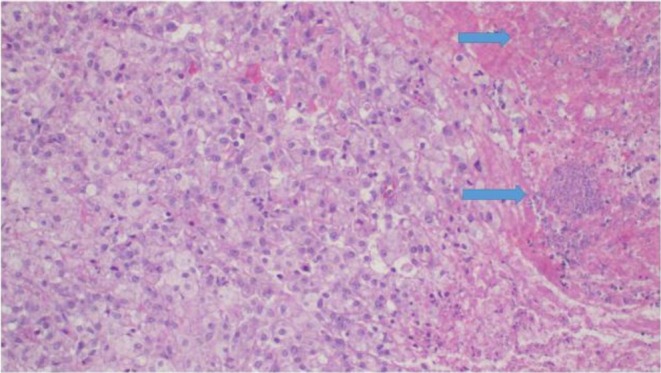
Photomicroscopy from salpingo‐oophorectomy specimen demonstrates diffuse infiltration of foamy macrophages, histiocytes, scattered lymphocytes, plasma cells, and areas of necrosis (blue arrows), H&E staining at 20× original magnification.

The postoperative recovery was uneventful. She was ready for discharge on postoperative Day 5. The family was counseled about the risk of recurrence and future fertility implications. Macroscopic examination of the specimen consisted of fragments of tissue measuring 5 × 4 × 3 cm. The surface was white, firm, and friable. On the cut surface, the tissue appeared whitish‐yellow with areas of hard and friable or soft texture. Microscopically, it demonstrated a diffuse cellular population of foamy histiocytes, fibroblasts, scattered multinucleated giant cells, plasma cells, lymphocytes, and neutrophils, along with confluent zones of geographic necrosis (Figures 3 and 4). This morphology was consistent with xanthogranulomatous salpingo‐oophoritis. There was no evidence of malignancy. Her follow‐up visit was planned for 2 weeks after her discharge, at which point the child was recovering well. At her 2‐month follow‐up visit, the CT scan showed no recurrence of the mass, and the wound had fully healed.

## 5 Discussion

In this report, we discuss the rarity of this condition, particularly in the pediatric population, the literature surrounding similar reports, and the many challenges that arise in the management of xanthogranulomatous salpingo‐oophoritis. Diagnosing an exceedingly rare condition such as this requires knowledge of the condition and a level of clinical suspicion when met with unusual and inconsistent clinical presentations. We hope that documentation of this case in the literature will support clinicians in diagnosing this condition and thereby enable them to provide high‐quality, appropriate care.

Xanthogranulomatous salpingo‐oophoritis is an exceedingly rare inflammatory condition with no previously reported cases in sub‐Saharan Africa and only one previously reported case in the pediatric population [8]. Over recent years, there has been a growing body of reported cases of xanthogranulomatous oophoritis. Increasing awareness of this rare entity allows for more efficient diagnosis and effective management [6, 9]. While on average, xanthogranulomatous oophoritis tends to present between 25 and 75, few cases have been reported in adolescents and younger adults. For example, in 2018, Bhatnagar et al. reported a case of a 20‐year‐old female who presented with intermittent fever and abdominal pain. She was found to have a thick‐walled, cystic adnexal mass with partial septations measuring 8.0 × 4.6 × 4.4 cm. Following an exploratory laparotomy and right oophorectomy, histolopathological examination of the ovarian tissue revealed sheets and nodules of foamy macrophages, histiocytes, lymphocytes, neutrophils, and dense fibrosis, confirming the diagnosis of xanthogranulomatous oophoritis [9]. To the best of our knowledge, this case represents the second youngest documented case of the disease. The rarity of this case is compounded by the location of the xanthogranulomatous inflammation and the absence of a history suggestive of pelvic inflammatory disease [10].

While the exact etiology of xanthogranulomatous oophoritis is not well understood, a number of potential mechanisms have been proposed, including infection, endometriosis, intrauterine contraceptive device, or pelvic inflammatory disease. In this patient, the precise cause of the condition could not be identified. However, given her age, lack of menarche, and lack of sexual activity, infection seems to be the most likely etiology. Various microorganisms, including *Bacteroides fragilis*, *Escherichia coli*, *Staphylococcus aureus* , and *Salmonella typhi* , have been implicated in the pathogenesis of xanthogranulomatous oophoritis [1]. Given the age of our patient at presentation, the possibility of an inborn error of metabolism should be given due credence. Unfortunately, testing for these inherited conditions is not something that is widely available in Tanzania, and there is no routine screening for these conditions at birth.

Additionally, in 2017, Jindal et al. reported a case of xanthogranulomatous inflammation of the ovary in a 17‐year‐old female who presented with abdominal pain and intermittent fever. Imaging studies revealed a large solid cystic pelvic mass with internal septations, and she had a slightly elevated CA‐125 on laboratory results. Following surgical removal of the mass, histopathology showed sheets of foamy macrophages and lymphocytic‐plasmocytic and eosinophilic infiltrate [11]. This case presented a diagnostic dilemma for the team involved in this patient's care. First, the patient's clinical presentation with abdominal distention and pain, as well as the 13 × 13 × 10 cm mass identified on CT scan and lymphadenopathy was highly suggestive of malignancy. Second, her elevated alpha fetal protein (AFP) further pointed to a diagnosis of malignancy, as AFP is commonly elevated in patients with yolk sac tumors. However, while AFP is a sensitive marker for these tumors, it is unspecific and can be elevated in a number of other conditions, including liver disease, pregnancy, and certain infections. Finally, the presence of pulmonary nodules on lung imaging raised suspicion for pulmonary metastases, further supporting a potential diagnosis of malignancy.

As described, the differential diagnoses for xanthogranulomatous salpingo‐oophritis can include chronic infections such as tuberculosis and malignancies such as yolk sac tumors, dysgerminomas, germ cell tumors, and neuroblastomas. Of note, our patient had an elevated AFP and a normal CA‐125 level. Whilst these results are highly nonspecific, they would support the diagnosis of a hormone‐secreting tumor without peritoneal irritation.

Given the clinical symptoms, imaging studies, and laboratory values, a diagnosis of malignancy was presumed, and she was treated with chemotherapy. It is standard practice to confirm the diagnosis of ovarian malignancy histologically before administering chemotherapy. However, in cases in which biopsy is not feasible, as in this case, empiric chemotherapy can be considered. This is especially true in resource‐limited settings where there may be financial or supply chain barriers to accessing diagnostic tests. The use of chemotherapy prior to tissue diagnosis presents a number of clinical and ethical challenges. Empiric chemotherapy without histological confirmation of malignancy exposes patients to unnecessary toxicity, potential organ damage, and delays in appropriate surgical management of the condition. For this reason, it is important to consider benign conditions, such as xanthogranulomatous oophoritis, when presented with a patient with an adnexal mass. This case highlights the importance of prioritizing histological diagnosis prior to systemic therapy, particularly in young patients who may desire future fertility.

The weaknesses of our case are clear and we hope will provide instructive learning points for clinicians facing similar diagnostic dilemmas in the future. Perhaps the most significant weakness was the initial misdiagnosis and subsequent unnecessary treatment. The issue of suspecting and potentially misdiagnosing xanthogranulomatous salpingo‐oophritis as a pelvic malignancy is well documented in the literature, [12, 13]; diagnosing exceedingly rare conditions requires prior knowledge of the condition and at least some level of clinical suspicion. Unfortunately, our misdiagnosis was acted upon before the correct diagnosis was made; the result was an 8‐year‐old child receiving 5 cycles of chemotherapy and systemic antibiotic therapy. Regrettably, it is possible that this may have consequences for development and future fertility.

There are several learning points to take from this case. Perhaps the most pertinent is that unusual presentations require thorough consideration of a broad list of rare differentials. Furthermore, before treatment is started, all efforts should be made to make a histopathological diagnosis. A recent study in the literature supported the use of contrast‐enhanced MRI to help clinicians differentiate xanthogranulomatous salpingo‐oophritis from malignancy [12]; this is not something that would be widely applicable to resource‐poor settings such as our own. We hope that the addition of this case to the literature will encourage clinicians to consider the diagnosis of xanthogranulomatous salpingo‐oophritis in the future. Furthermore, young patients who have received chemotherapy should receive long‐term follow‐up to assess for the late effects of systemic chemotherapy.

## 6 Conclusion

This case of a pediatric patient with xanthogranulomatous salpingo‐oophoritis in Tanzania adds to the limited body of literature on the condition and represents, to the best of our knowledge, the first reported pediatric case from sub‐Saharan Africa. This case study underscores the importance of preoperative histopathology that plays a critical role in the management of suspected neoplastic lesions. Histopathology helps prevent misdiagnosis, thus avoiding unnecessary chemotherapy and surgery, and it ensures appropriate and tailored management. It highlights the importance of considering this rare benign condition when presented with a patient with an adnexal mass, particularly in areas with a high prevalence of infectious and inflammatory diseases. Finally, this case emphasizes the importance of multidisciplinary management of cases of adnexal masses in pediatric patients. Involving a diverse treatment team can facilitate accurate diagnosis, guide optimal surgical intervention, and optimize fertility preservation while simultaneously preventing unnecessary morbidity. We engaged a team of gynecologists, oncologists, and pathologists in the care of this patient, utilizing the expertise of multiple fields in making treatment decisions for this rare condition.

## Author Contributions

**John Lugata:** conceptualization, data curation, methodology, project administration, resources, validation, writing – original draft, writing – review and editing. **Kristen Riley:** conceptualization, data curation, methodology, project administration, resources, validation, writing – original draft, writing – review and editing. **Laetitia Makower:** conceptualization, data curation, methodology, project administration, resources, validation, writing – original draft, writing – review and editing. **Baraka Shao:** conceptualization, data curation, methodology, project administration, resources, validation, writing – original draft, writing – review and editing. **Tecla Lyamuya:** conceptualization, data curation, methodology, project administration, resources, validation, writing – original draft, writing – review and editing. **Alex Mremi:** conceptualization, data curation, methodology, project administration, resources, validation, writing – original draft, writing – review and editing.

## Ethics Statement

The patient provided written informed consent to allow for her de‐identified medical information to be used in this publication. A waiver for ethical approval was obtained from the authors' institution review board committee.

## Consent

Written informed consent for the publication of clinical details and images was obtained from the patient. A copy of the consent is available for review by the chief editor of this journal.

## Conflicts of Interest

The authors declare no conflicts of interest.

## Data Availability

No data generated from this study.
